# Suppression of skin tumorigenesis in CD109-deficient mice

**DOI:** 10.18632/oncotarget.12653

**Published:** 2016-10-14

**Authors:** Masaki Sunagawa, Shinji Mii, Atsushi Enomoto, Takuya Kato, Yoshiki Murakumo, Yukihiro Shiraki, Naoya Asai, Masato Asai, Masato Nagino, Masahide Takahashi

**Affiliations:** ^1^ Department of Pathology, Nagoya University Graduate School of Medicine, Nagoya, Japan; ^2^ Division of Surgical Oncology, Department of Surgery, Nagoya University Graduate School of Medicine, Nagoya, Japan; ^3^ Division of Molecular Pathology, Center for Neurological Disease and Cancer, Nagoya University Graduate School of Medicine, Nagoya, Japan; ^4^ Tumour Cell Biology Laboratory, The Francis Crick Institute, Lincoln's Inn Fields Laboratories, London, United Kingdom; ^5^ Department of Pathology, Kitasato University School of Medicine, Sagamihara, Japan

**Keywords:** skin carcinogenesis, CD109, TGF-β, p21, Nrf2

## Abstract

CD109 is a glycosylphosphatidylinositol-anchored glycoprotein that is highly expressed in several types of human cancers, particularly squamous cell carcinomas. We previously reported that CD109-deficient mice exhibit epidermal hyperplasia and chronic skin inflammation. Although we found that CD109 regulates differentiation of keratinocytes *in vivo*, the function of CD109 in tumorigenesis remains unknown. In this study, we investigated the role of CD109 in skin tumorigenesis using a two-stage carcinogenesis model in CD109-deficient mice with chronic skin inflammation. Immunohistochemical analysis revealed a higher level of TGF-β protein expression in the dermis of CD109-deficient mice than in that of wild-type mice. Additionally, immunofluorescence analysis showed that Smad2 phosphorylation and Nrf2 expression were enhanced in primary keratinocytes from CD109-deficient mice compared with in those from wild-type mice. Although no significant difference was found in conversion rates from papilloma to carcinoma between wild-type and CD109-deficient mice in the carcinogenesis model, we observed fewer and smaller papillomas in CD109-deficient mice than in wild-type mice. Apoptosis and DNA damage marker levels were significantly reduced in CD109-deficient skin compared with in wild-type skin at 24 h after 7, 12-dimethylbenz (α) anthracene treatment. Furthermore, mutation-specific PCR revealed that the mutation frequency of the H-*ras* gene was less in CD109-deficient skin than in wild-type skin in this model. These results suggest that CD109 deficiency suppresses skin tumorigenesis by enhancing TGF-β/Smad/Nrf2 pathway activity and decreasing the mutation frequency of the H-*ras* gene.

## INTRODUCTION

Squamous cell carcinoma (SCC) is the second most common skin cancer, with a rising incidence over the past three decades. Approximately 700,000 new cases of cutaneous SCC are diagnosed annually in the United States of America [1]. Although skin SCCs have a generally favorable prognosis, approximately 1.5%–2% of patients die from the disease [1, 2]. Additionally, skin SCCs are most commonly found in sun-exposed areas of the body, such as the head and neck, where surgery may be disfiguring [2]. Several risk factors are associated with skin SCC, including exposure to ultraviolet light, arsenic, or tobacco; human papilloma virus infection; and exposure to chemicals, including 7, 12-dimethylbenz (α) anthracene (DMBA) [3, 4].

CD109, a glycosylphosphatidylinositol-anchored glycoprotein, is a member of the α_2_-macroglobulin/C3, C4, C5 family of thioester-containing proteins [5–8]. CD109 is a cell surface protein expressed on CD34-positive bone marrow mononuclear cells, activated T lymphoblasts, activated platelets, endothelial cells and mesenchymal stem cell subsets [5, 9–11]. We previously reported high levels of CD109 expression in various tumor cell lines and tumor tissues including SCCs of the lung, esophagus, uterus and oral cavity; malignant melanoma of the skin; and urothelial carcinoma of the urinary bladder [12–20]. CD109 expression was significantly higher in well-differentiated SCCs of the oral cavity than in normal oral mucosa and moderately or poorly differentiated SCCs [17, 20]. These findings suggest that CD109 is associated with tumor development, especially in SCCs. CD109 also functions as a negative regulator of transforming growth factor (TGF)-β signaling in human keratinocytes. CD109 inhibits receptor-regulated Smad (R-Smad) activation, probably by direct modulation of TGF-β receptor activity [21–24]. CD109 is also reportedly associated with human psoriasis [25, 26], and CD109-deficient mice have been shown to exhibit inflammatory cell infiltration of the dermis [27]. The majority of infiltrating cells were found to be T lymphocytes, which were identified as CD3-positive by immunohistochemistry.

Signaling via the TGF-β receptor system induces a wide range of biological responses including cell proliferation, differentiation, migration and apoptosis; tissue remodeling; and immune response [28, 29]. Ligand-mediated assembly of TGF-β receptor (TGFBR) I and TGFBRII initiates an intracellular phosphorylation cascade. Activated TGFBRII transphosphorylates TGFBRI, which subsequently phosphorylates R-Smads such as Smad2/3, which in turn enables the R-Smads to bind a common mediator, Smad4. R-Smad/Smad4 complexes accumulate in the nucleus where they act as transcription factors for target genes [28]. TGF-β functions as a tumor suppressor in precancerous cells, but as an enhancer of invasion and metastasis in more advanced carcinoma cells [29, 30]. In addition, a recent report showed that TGF-β activates p21^WAF1/CIP1^ (p21) and nuclear factor erythroid 2-related factor 2 (Nrf2), thereby enhancing glutathione metabolism and antioxidant response in SCCs *in vivo* [31]. p21 prevents cell cycle progression [31] and activates the Nrf2 transcription factor [32]. Nrf2-deficient mice were found to be more susceptible to skin tumorigenesis [33] and Nrf2 activation protected keratinocytes in the early phase of skin tumorigenesis [34].

We used one of the most frequently used carcinogenesis models, in which topical application of DMBA, a polycyclic aromatic hydrocarbon that induces DNA alteration, was followed by the topical application of tetradecanoyl-phorbol acetate (TPA), which stimulates inflammation and epidermal proliferation [35]. In a previous study using this model, H-*ras^(Q61L)^* mutation, shown to be associated with H-*ras* gene amplification, was detected in papillomas as well as carcinomas [36]. This observation suggests that H-*ras* is an early driver in the carcinogenesis model [35, 36]. In this study, we investigated the role of CD109 in the development of SCC using CD109-deficient mice.

## RESULTS

### CD109 deficiency enhances TGF-β signaling in mouse skin

**Figure 1:**
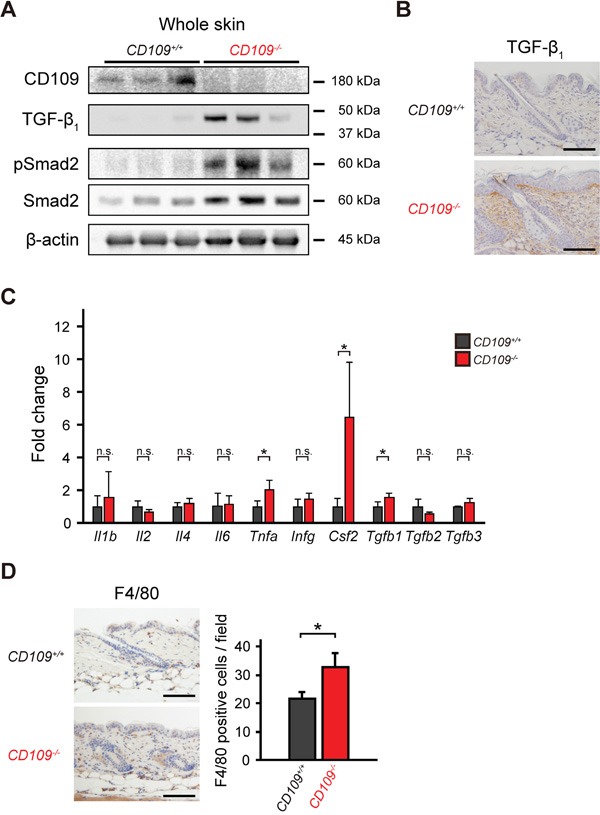
CD109 deficiency enhanced TGF-β signaling and induced macrophage infiltration in mouse skin **A.** Immunoblot analysis of whole-skin lysates from 6-week-old *CD109^+/+^* and *CD109^−/−^* mice using antibodies against CD109, TGF-β1, pSmad2, and Smad2 (n = 3 per group). Expression of β-actin is shown as a loading control. **B.** Immunohistochemical analysis of TGF-β1 expression in the dermis of 6-week-old *CD109^−/−^* and *CD109^+/+^* mice. Scale bars, 100 μm. **C.** Expression levels of mRNAs encoding the indicated cytokines in the skin of 6-week-old *CD109^+/+^* and *CD109^−/−^* mice, determined by quantitative PCR (n = 4 per group). **D.** Immunohistochemical analysis showing F4/80-positive cells (macrophages) in the skin of 6-week-old *CD109^+/+^* and *CD109^−/−^* mice. Right, quantification of F4/80-positive cells in the dermis. Scale bars, 100 μm; n.s., not significant; *P < 0.05.

Although CD109 is reported to function as a negative regulator of TGF-β signaling in human keratinocytes [22–25], we previously observed no effect of CD109 deficiency on the TGF-β signaling pathway in the mouse epidermis *in vivo* [27]. In this study, we first examined TGF-β/Smad signaling in the whole skin of *CD109^+/+^* and *CD109^−/−^* mice. Immunoblot analysis using lysates prepared from the whole skin of *CD109^+/+^* and *CD109^−/−^* mice showed that the TGF-β/Smad signaling pathway was activated in *CD109^−/−^* skin but not in *CD109^+/+^* skin (Figure 1A). Immunohistochemical analysis revealed a higher level of TGF-β1 protein in the dermis of *CD109^−/−^* mice than in that of *CD109^+/+^* mice (Figure 1B). TGF-β1 was undetectable in the epidermis of both *CD109^+/+^* and *CD109^−/−^* mice; the epidermis–dermis junction was more strongly stained with anti-TGF-β1 antibody than the deeper dermis in *CD109^−/−^* skin. Conversely, no apparent differences in TGFBRI or TGFBRII expression were observed immunohistochemically between *CD109^+/+^* and *CD109^−/−^* skin (Supplementary Figure S1A).

We next investigated the expression of inflammation-related genes, including *Tgfb1*, in the skin of *CD109^+/+^* and *CD109^−/−^* mice. We used Q-PCR analysis to determine the mRNA expression levels of various cytokines (Figure 1C). There were no significant differences in mRNA levels of several cytokines, including interleukins and interferon-γ, between *CD109^+/+^* and *CD109^−/−^* mice; however *Tgfb1*, *Csf2*, and *Tnfa* mRNA expression levels were significantly higher in *CD109^−/−^* mice than in *CD109^+/+^* mice (*P* < 0.05). In addition to the increase observed in TGF-β1 protein levels (Figure 1B), the protein levels of TNF-α and GM-CSF (encoded by *Csf2*) were higher in the skin of *CD109^−/−^* mice than in that of *CD109^+/+^* mice (Supplementary Figure S1B). GM-CSF is produced by various cells, including macrophages and fibroblasts; it activates macrophages and induces TNF-α production [37]. Immunohistochemical analysis using anti-F4/80 antibody revealed more macrophages infiltrating the dermis of *CD109^−/−^* mice than that of *CD109^+/+^* mice (Figure 1D). These findings indicate that CD109 deficiency not only enhances TGF-β signaling but also induces macrophage infiltration in mouse skin.

To clarify the relationship between the increase in TGF-β1 levels and the increase in the number of macrophages infiltrating the dermis in CD109-deficient mice, we performed macrophage depletion experiments using clodronate liposomes. Macrophage depletion by subcutaneous injection of clodronate lipsomes was confirmed by immunohistochemical analysis with anti-F4/80 antibody (Supplementary Figure S2A). There were no significant differences in the number of F4/80-positive cells between clodronate-treated *CD109^+/+^* and *CD109^−/−^* skin (Supplementary Figure S2A). Additionally, no significant differences were observed in mRNA levels of *Tgfb1*, *Tnfa* and *Csf2* between clodronate-treated *CD109^+/+^* and *CD109^−/−^* skin (Supplementary Figure S2B). These findings suggest that TGF-β1 was at least partly secreted from the macrophages that infiltrated the dermis in CD109-deficient mice.

### Roles of CD109 in DMBA-induced cytotoxicity in primary mouse keratinocytes

**Figure 2:**
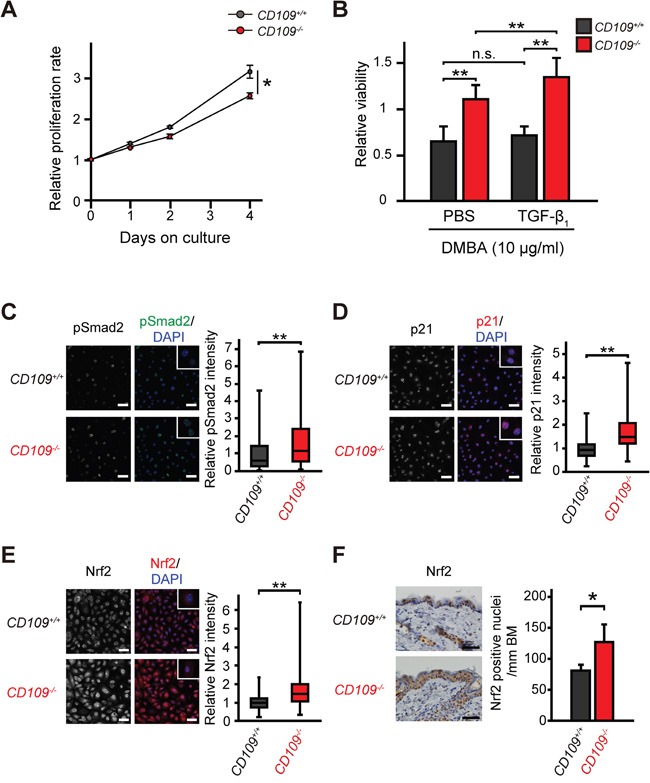
Cellular characteristics of primary mouse keratinocytes **A.** *CD109^+/+^* and *CD109^−/−^* keratinocyte proliferation rates, measured by WST-1 assay (n = 6 per group). **B.** Viability of *CD109^+/+^* and *CD109^−/−^* keratinocytes in the presence of DMBA (10 μg/ml), measured by WST-1 assay (n = 3 per group). Cells were pretreated with PBS only (control) or TGF-β1 (10 ng/ml) for 24 h before incubation with DMBA. **C-E.** Fluorescence immunostaining for pSmad2 (C), p21 (D), and Nrf2 (E) in *CD109^+/+^* and *CD109^−/−^* keratinocytes (n = 90 per group). Insets show higher magnification of positive cells. Scale bars, 50 μm. Right graphs, quantification of immunofluorescence intensity. **F.** Immunohistochemical staining for Nrf2 in the skin of 6-week-old *CD109^+/+^* and *CD109^−/−^* mice (left panels). The number of Nrf2-positive cells in epidermis was counted per mm of basement membrane (BM) (right graph; n = 4 per group). DMBA, 7, 12-dimethylbenz (α) anthracene; *P < 0.05; **P < 0.01.

A recent study demonstrated that TGF-β enhanced glutathione metabolism and antioxidant response in SCCs via activation of p21 and Nrf2 [31]. To examine the effect of CD109 deficiency on the antioxidant response in keratinocytes, we assessed proliferation rate and viability of primary mouse keratinocytes by WST-1 assay. The proliferation rate of *CD109^+/+^* keratinocytes was significantly higher than that of *CD109^−/−^* keratinocytes in the defined medium without TGF-β (*P* < 0.05; Figure 2A). This finding is consistent with a previous study that showed that CD109-overexpressing HEK293 cells grew faster than control cells [17]. Interestingly, the viability of *CD109^−/−^* keratinocytes was significantly higher than that of *CD109^+/+^* keratinocytes in the presence of DMBA or cisplatin (CDDP), regardless of TGF-β1 pretreatment (*P* < 0.01; Figure 2B, *P* < 0.05; Supplementary Figure S3A). No significant differences were observed in cell viability in the presence of DMBA between TGF-β1-pretreated and PBS-pretreated *CD109^+/+^* keratinocytes (Figure 2B, Supplementary Figure S3B); however, the viability of *CD109^−/−^* keratinocytes in the presence of DMBA was significantly higher after TGF-β1-pretreatment than after PBS-pretreatment (*P* < 0.01; Figure 2B, *P* < 0.05; Supplementary Figure S3C). Additionally, the viability of *CD109^−/−^* keratinocytes in the presence of DMBA was decreased by p21 knockdown using siRNA targeting *p21* (Supplementary Figure S3D and S3E). Moreover, the viability of *CD109^−/−^* keratinocytes pretreated with TGFBRI inhibitor was significantly lower than that of PBS-pretreated *CD109^−/−^* keratinocytes in the presence of DMBA (*P* < 0.01; Supplementary Figure S3C). This finding suggests that resistance of TGF-β to DMBA- or CDDP-induced cytotoxicity was enhanced in *CD109^−/−^* keratinocytes compared with in *CD109^+/+^* keratinocytes. Immunofluorescence analysis showed that Smad2 phosphorylation was elevated in *CD109^−/−^* keratinocytes compared with in *CD109^+/+^* keratinocytes (Figure 2C).

To investigate the mechanism underlying the increased viability of *CD109^−/−^* keratinocytes in the presence of DMBA or CDDP, we analyzed the expression levels of p21 and Nrf2, mediators of the antioxidant response, which are known to protect against toxic effects of DMBA and CDDP [31, 38]. Immunofluorescence analysis showed that nuclear localization of p21 and Nrf2 was significantly increased in *CD109^−/−^* keratinocytes compared with in *CD109^+/+^* keratinocytes (*P* < 0.01; Figure 2D, 2E). We also examined Nrf2 expression levels *in vivo* using immunohistochemistry, and observed higher Nrf2 expression in the epidermis of 6-week-old *CD109^−/−^* mice than it that of *CD109^+/+^* mice (Figure 2F). In addition, we evaluated detoxification enzymes, some of which are regulated by Nrf2 in keratinocytes [39]. There were no significant differences in the expression levels of detoxification enzymes that are reportedly not regulated by Nrf2 between *CD109^+/+^* and *CD109^−/−^* skins (Supplementary Figure S4A); however, the mRNA and protein levels of several detoxification enzymes that are reportedly regulated by Nrf2 [34, 40] were elevated in *CD109^−/−^* skin compared with in *CD109^+/+^* skin (Supplementary Figure S4B and S4C). Moreover, microarray analysis revealed that Akr1c19, one of the Nrf2-related detoxification enzymes [40], was downregulated in *CD109^+/+^* skin and upregulated in *CD109^−/−^* skin after DMBA treatment, as compared with untreated skin (Supplementary Tables S2 and S3). These findings suggest that CD109 deficiency increases resistance to DMBA- or CDDP-induced cytotoxicity in primary mouse keratinocytes via the TGF-β/p21/Nrf2 pathway.

### CD109 deficiency suppresses DMBA/TPA-induced skin tumorigenesis

**Figure 3:**
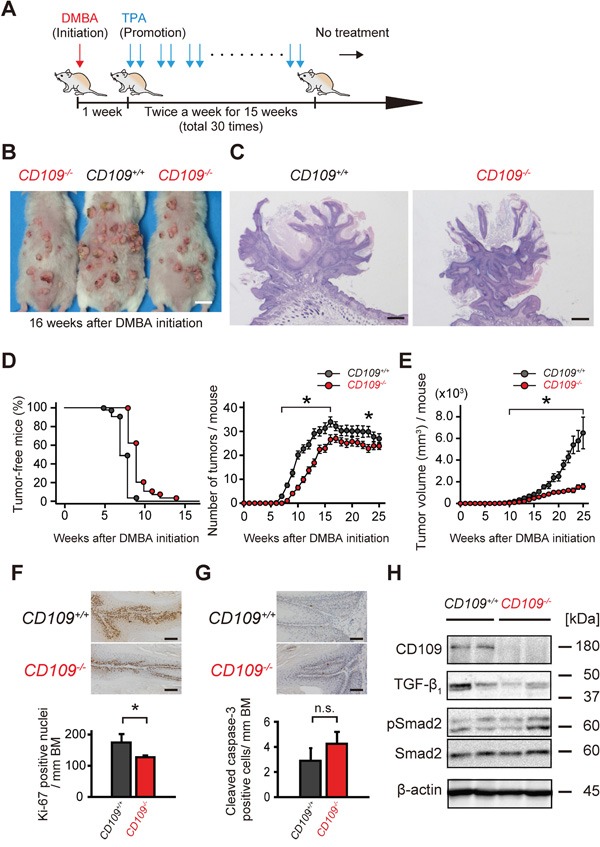
DMBA/TPA-induced skin tumorigenesis was suppressed in *CD109^−/−^* mice **A.** Schematic diagram of the two-stage chemical carcinogenesis protocol in mouse skin. **B.** Representative macroscopic images of *CD109^+/+^* and *CD109^−/−^* mice at 16 weeks after DMBA initiation. Scale bars, 1 cm. **C.** Representative histological images of papillomas isolated from *CD109^+/+^* and *CD109^−/−^* mice at 17 weeks after DMBA initiation. Scale bars, 500 μm. **D.** Percentage of tumor-free mice (left) and average number of tumors per mouse (right) during DMBA/TPA tumorigenesis (27 mice in the *CD109^+/+^* group and 22 mice in the *CD109^−/−^* group). **E.** Tumor volume was significantly decreased in *CD109^−/−^* mice compared with in *CD109^+/+^* mice 10–25 weeks after DMBA initiation (n = 9 per group). **F, G.** Immunohistochemical staining for Ki-67 (F) and cleaved caspase-3 (G) in *CD109^+/+^* and *CD109^−/−^* papillomas at 17 weeks after DMBA initiation (left). The numbers of positive cells in epidermis were counted per mm of basement membrane (BM) (right, n = 6 per group). Scale bars, 100 μm. **H.** Immunoblot analysis of *CD109^+/+^* and *CD109^−/−^* papilloma lysates prepared at 17 weeks after DMBA initiation, using antibodies against CD109, TGF-β1, pSmad2, and Smad2. β-actin expression is shown as a loading control. TPA, 12-*O*-tetradecanoylphorbol-13-acetate; n.s., not significant; *P < 0.05.

To examine the role of CD109 in skin tumorigenesis *in vivo*, we used a two-stage carcinogenesis model in CD109-deficient mice with chronic skin inflammation. A schematic diagram of the two-stage carcinogenesis protocol using DMBA and TPA is shown in Figure 3A. Macroscopically, we found fewer and smaller skin tumors in *CD109^−/−^* mice compared with in *CD109^+/+^* mice in this model (Figure 3B). The tumors were diagnosed histologically as papillomas at 17 weeks after DMBA initiation. There were no apparent histological differences between *CD109^+/+^* and *CD109^−/−^* papillomas, except for the tumor size (Figure 3C, Supplementary Figure S5A). Skin tumors were counted and measured once a week to quantify the tumor number and size. Skin tumors tended to appear later and tumor number per mouse was significantly lower in *CD109^−/−^* mice than in *CD109^+/+^* mice (*P* < 0.05; Figure 3D). Total tumor volume per mouse was also significantly lower in *CD109^−/−^* mice than in *CD109^+/+^* mice (*P* < 0.05; Figure 3E).

Epidermal basal cell proliferation and keratinocyte apoptosis in papillomas were assessed immunohistochemically by staining with anti-Ki-67 and anti-cleaved caspase-3 antibodies, respectively. The number of Ki-67-positive cells was significantly higher in papillomas of *CD109^+/+^* mice than in those of *CD109^−/−^* mice at 17 weeks after DMBA initiation (*P* < 0.05; Figure 3F, Supplementary Figure S5B). However, no significant difference in the number of cleaved caspase-3-positive cells was detected between the two mouse groups (Figure 3G, Supplementary Figure S5C).

To address the mechanism of increased resistance to DMBA/TPA-induced tumorigenesis in *CD109^−/−^* skin, lysates from the papillomas were subjected to immunoblot analysis using antibodies against TGF-β1, phospho-Smad2 and Smad2 (Figure 3H). The results show that there were no apparent changes in the levels of TGF-β1 expression or Smad2 phosphorylation between *CD109^+/+^* and *CD109^−/−^* papillomas.

We sacrificed the mice at 25 or 40 weeks after DMBA initiation and performed pathological analyses (Table 1, Supplementary Table S4). Our study did not show any correlation between CD109 deficiency and pathological features of invasive tumors (SCCs), such as malignant conversion rate, histological differentiation (Supplementary Figure S5D), invasion depth (Table 1, Supplementary Table S4) or number of metastatic lesions (Supplementary Figure S5E). These findings suggest that CD109 deficiency suppresses squamous cell papilloma formation *in vivo*, and that the chronic skin inflammation observed in *CD109^−/−^* mice with increased *Tgfb1*, *Csf2*, and *Tnfa* expression may reduce early-stage tumorigenesis, but not malignant conversion.

**Table 1:** Pathological analysis of skin tumors from *CD109^+/+^* and *CD109^−/−^* mice at 25 weeks after DMBA initiation

*CD109* genotypes	+/+	−/−	*P* value
Number of mice	9	9	
Total number of tumors	258	193	
Total number of invasive tumors	36	31	
Malignant conversion rate^a)^	14.0%	16.1%	0.53
**Primary skin lesions**			
Invasive tumor size (μm)	6.9 ± 0.7	5.8 ± 0.5	0.20
Differentiation			0.57
Conventional SCCs^b)^			
Well differentiated	26 (72%)	20 (65%)	
Moderately differentiated	6 (17%)	9 (29%)	
Poorly differentiated	3 (8%)	1 (3%)	
Spindle cell carcinoma	1 (3%)	1 (3%)	
Invasion depth			0.43
Dermis	32 (89%)	30 (97%)	
Hypodermis	3 (8%)	1 (3%)	
Muscle	1 (3%)	0 (0%)	
**Metastatic lesions**	2/9 (22%)	0/9 (0%)	0.13

a) Malignant conversion rate = total number of invasive tumors/total number of tumors

b) SCCs, squamous cell carcinomas

### DMBA/TPA-induced epidermal proliferation is suppressed in *CD109^−/−^* mice

**Figure 4:**
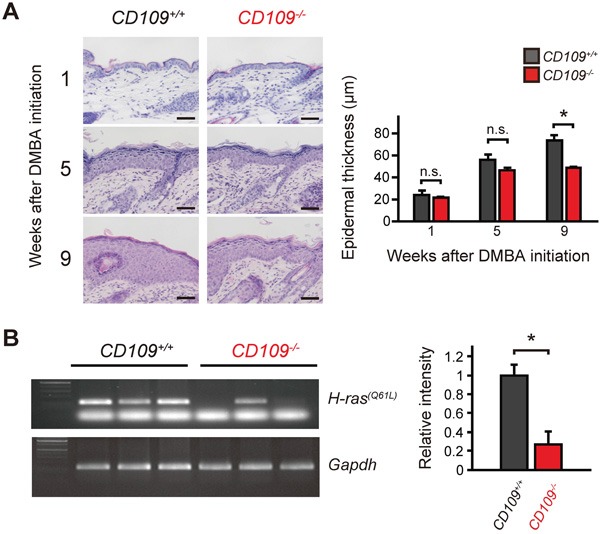
DMBA/TPA-induced proliferation of epidermal keratinocytes was suppressed in *CD109^−/−^* mice **A.** Left, representative microscopic images of *CD109^+/+^* and *CD109^−/−^* skins treated with DMBA alone (1 week after DMBA initiation) or DMBA and TPA (5 and 9 weeks after DMBA initiation). Right, quantification of epidermal thickness at indicated time points in *CD109^+/+^* and *CD109^−/−^* skins (n = 4 per group). Scale bars, 50 μm. **B.** H-*ras^(Q61L)^* mutation-specific PCR assay using genomic DNA from *CD109^+/+^* and *CD109^−/−^* skins at 9 weeks after DMBA initiation (left). Right, quantification of signal intensity of mutant H-*ras^(Q61L)^* normalized to that of *Gapdh* (n = 3 per group). n.s., not significant; *P < 0.05.

We next compared the histological changes in dorsal skin before papilloma formation between *CD109^+/+^* and *CD109^−/−^* mice. Compared with that of *CD109^−/−^* mice, the skin of *CD109^+/+^* mice exhibited apparent thickening of the epidermis at 9 weeks after DMBA initiation (Figure 4A). We also performed immunohistochemical analysis using antibodies against TGF-β1 and Nrf2. TGF-β1 expression levels tended to be higher in *CD109^−/−^* skin than in *CD109^+/+^* skin at each time point, but no significant differences were observed in Nrf2 staining at 5 or 9 weeks after DMBA initiation (Supplementary Figure S6).

### H-*ras* mutation induced by DMBA/TPA treatment is less frequent in *CD109^−/−^* skin than in *CD109^+/+^ skin*

This finding prompted us to examine genetic changes before papilloma formation. Because H-*ras^(Q61L)^* mutation was reportedly found before the appearance of papillomas [36], we assessed H-*ras^(Q61L)^* gene mutation in skin using a mutation-specific PCR assay at 9 weeks after DMBA initiation. At this time point, no or few papillomas were observed in skin (Figure 3D). In this assay, less frequent mutation of the H-*ras^(Q61L)^* gene was detected in genomic DNA from *CD109^−/−^* skins compared with in that from *CD109^+/+^* skins (Figure 4B). We also investigated the effects of CD109 deficiency on epidermal proliferation with TPA treatment alone using a TPA-induced skin inflammation model (Supplementary Figure S7A); however, we identified no significant differences between *CD109^+/+^* and *CD109^−/−^* mice in epidermal thickness, the number of Ki-67-positive cells or inflammation-related gene expression levels (Supplementary Figure S7B–D). These results suggest that CD109 plays a role in the earlier phase of skin tumorigenesis, such as in DMBA-induced H-*ras^(Q61L)^* mutation.

### CD109 deficiency decreases skin sensitivity to DMBA

**Figure 5:**
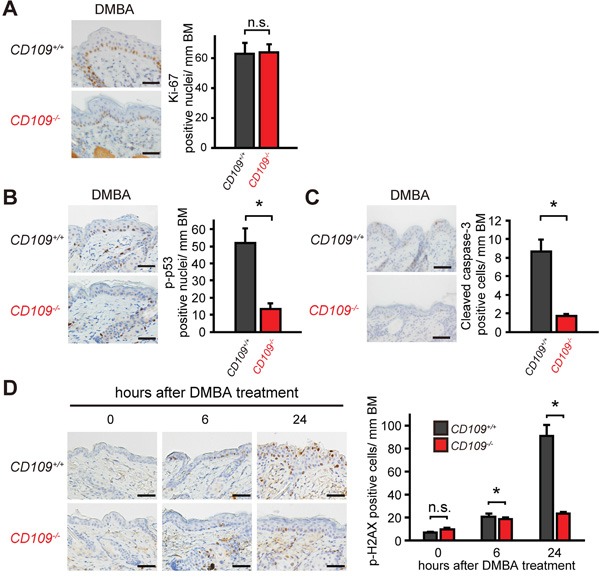
CD109 deficiency decreased skin sensitivity to DMBA **A-C.** Immunohistochemical staining for Ki-67 (A), p-p53 (B), and cleaved caspase-3 (C) in *CD109^+/+^* and *CD109^−/−^* skins at 24 h after DMBA treatment (left panels). The numbers of positive cells in epidermis were counted per mm of basement membrane (BM) (right graphs; n = 4 per group). Scale bars, 100 μm. **D.** Immunohistochemical staining for p-H2AX in *CD109^+/+^* and *CD109^−/−^* skins at 0, 6 and 24 h after DMBA treatment (left panels). The number of p-H2AX-positive cells in epidermis was counted per mm of BM (right graph; n = 4 per group). Scale bars, 100 μm. n.s., not significant; *P < 0.05.

To investigate the role of CD109 in the skin response to DMBA, we performed immunohistochemical analysis of *CD109^+/+^* and *CD109^−/−^* skins at 24 h after DMBA treatment (Figure 5). Although there was no significant difference in the number of Ki-67-positive cells between *CD109^+/+^* and *CD109^−/−^* skins, the number of cells positive for phospho-p53 as a cellular stress marker [41], cleaved caspase-3 as an apoptosis marker, and phospho-H2AX as a DNA damage marker [42] were reduced in *CD109^−/−^* skins compared with *CD109^+/+^* skins. These results suggest that CD109 deficiency decreases skin sensitivity to cellular stress including DNA damage by DMBA, resulting in less frequent mutation of the H-*ras^(Q61L)^* gene.

## DISCUSSION

We previously reported that CD109 is associated with human tumor development, especially in SCCs [12–14, 17, 20]. In this study, we therefore investigated the role of CD109 in skin tumorigenesis *in vivo*, using a two-stage carcinogenesis model in CD109-deficient mice. The number and size of papillomas were significantly decreased in *CD109^−/−^* mice compared with in *CD109^+/+^* mice; however, we found no significant differences in malignant conversion rate, histological type or invasion/metastasis between *CD109^+/+^* and *CD109^−/−^* tumors. Our data suggest that the chronic skin inflammation with TGF-β protein expression in the dermis that is observed in *CD109^−/−^* mice reduces early-stage skin tumorigenesis, but does not influence malignant conversion. Although the mechanisms underlying the development and progression of the corresponding human skin disease remain elusive, CD109 deficiency may be a factor in the tumor-suppressing effects of chronic skin inflammation.

While CD109 is reported to function as a negative regulator of TGF-β signaling in human keratinocytes [22–25], we previously observed no effect of CD109 deficiency on the TGF-β signaling pathway in physiological and pathological conditions *in vivo* [27]. In this study, we showed that CD109 deficiency enhances TGF-β/Smad signaling in whole skin, including dermis, and Nrf2 expression in the epidermis in skin tumorigenesis. Recently, Oshimori et al. [31] reported that the TGF-β/p21/Nrf2 pathway plays an important role in antioxidant metabolism in skin SCC. TGF-β was detected in skin stroma and Smad2 phosphorylation was enhanced in some skin stromal cells. The authors also detected Nrf2 and p21 expression in TGF-β-responding tumor cells at the tumor–stromal interface [31]. In addition, Nrf2-deficient mice were reported to be more susceptible to DMBA-induced skin tumorigenesis [33] and Nrf2 activation protected keratinocytes in the early phase of skin tumorigenesis [34]. These results are consistent with our data, suggesting that CD109 deficiency increases DMBA metabolism via the TGF-β/p21/Nrf2 pathway and resistance to DMBA-induced H-*ras* mutation in mouse keratinocytes.

Although we performed microarray analysis of the skin of DMBA-untreated or -treated *CD109^+/+^* and *CD109^−/−^* mice, we did not detect any changes to suggest possible mechanisms for the effects observed other than the TGF-β/p21/Nrf2 pathway. However, it is interesting to note that altered expression of genes including *Tpsab1*, *Shh*, *S100a7a* and *Il20*, which are associated with tumorigenesis or psoriasis, was observed in the microarray analysis (Supplementary Tables S3 and S5). Altered expression of tryptase α1 and β1, encoded by *Tpsab1*, has been reported to be associated with SCC; however, it is controversial whether they are increased or decreased in SCC [43, 44]. Sonic hedgehog, encoded by *Shh*, is well known to be involved in basal cell carcinoma formation [45], but to our knowledge, there have been no reports implicating Shh in SCC carcinogenesis. S100A15, encoded by *S100a7a*, and IL-20 are known to be upregulated in psoriatic skin [46, 47], which is similar to the phenotype of CD109-deficient mice [27].

Interestingly, we detected more TGF-β protein in the dermis of *CD109^−/−^* mice than in that of *CD109^+/+^* mice. While CD109 is known to negatively regulate TGF-β signaling in human keratinocytes, probably by direct modulation of receptor activity [21–24], our finding implies that CD109 might also suppress TGF-β signaling via reduction of TGF-β in the dermis. Notably, we detected a strong TGF-β signal in the epidermis–dermis junction in *CD109^−/−^* skin by immunohistochemistry using anti-TGF-β antibody, whereas TGF-β was undetectable in the epidermis. This result suggests that the epidermal–dermal interaction, involving TGF-β signaling, could contribute to the suppression of DMBA/TPA-induced epidermal proliferation in *CD109^−/−^* mice. Additionally, the finding that Smad2 phosphorylation levels were similar between *CD109^+/+^* and *CD109^−/−^* papillomas could result from the lower amount of stroma found in papillomas compared with in normal skin. One limitation of this study is that it does not address the mechanism underlying the increase in TGF-β levels in *CD109^−/−^* dermis. Although speculative, it is interesting to consider that TGF-β in *CD109^−/−^* dermis may result from increased secretion of TGF-β by dermal stromal cells, such as fibroblasts or macrophages.

Although the epidermal thickness and BrdU-positive ratio (proliferation index) were significantly increased in the epidermis of *CD109^−/−^* mice compared with in that of *CD109^+/+^* mice without DMBA/TPA treatment in our previous study [27], the epidermal thickness and the number of Ki-67-positive cells was significantly decreased in DMBA/TPA-treated skin of *CD109^−/−^* mice compared with in that of *CD109^+/+^* mice in this study. This difference suggests that CD109 deficiency suppresses DMBA/TPA-induced epidermal hyperplasia or papilloma formation by a mechanism different from those operating under physiological conditions as observed in our previous study. In addition, we previously reported that CD109 expression levels were significantly higher in well-differentiated SCCs of the oral cavity and in low-grade urothelial carcinomas of the urinary bladder than in moderately or poorly differentiated SCCs and in high-grade urothelial carcinomas, respectively [17, 19, 20]. These results suggest that CD109 expression may be associated with tumor differentiation. However, we found no significant differences in the histological differentiation of carcinomas between *CD109^+/+^* and *CD109^−/−^* mice in the present study. Further investigation is necessary to clarify the role of CD109 in invasion, differentiation, and metastasis of skin cancer.

In conclusion, our findings suggest that CD109 deficiency plays a role in suppression of the mutation rate of the H-*ras* gene in the early phase of DMBA/TPA-induced skin tumorigenesis by enhancing the TGF-β/p21/Nrf2 pathway, and significantly reduces the number and size of papillomas. Further analyses using different types of tumorigenesis models such as ultraviolet radiation would provide insights into the mechanism of the tumor-suppressing effects of CD109 associated with chronic inflammation.

## MATERIALS AND METHODS

### Mice

Wild-type FVB/N (*CD109^+/+^*) mice were purchased from CLEA Japan, Inc. (Tokyo, Japan). *CD109^−/−^* mice were generated as previously described [27] and backcrossed onto the FVB/N background for 10 generations. FVB/N mice display a high incidence of SCC after treatment with various tumor-induction protocols [48]. All mice were housed in the animal facilities of Nagoya University Graduate School of Medicine. All animal protocols were approved by the Animal Care and Use Committee of Nagoya University Graduate School of Medicine (approval ID number 27338).

### RNA isolation and real-time quantitative PCR (Q-PCR)

Whole-skin samples were snap-frozen in liquid nitrogen and ground to a fine powder using Multi-Beads Shocker (Yasui Kikai Corporation, Osaka, Japan). Total RNA was isolated from cultured cells or tissue samples using TRIzol reagent (Invitrogen, Carlsbad, CA), treated with DNase I (Qiagen, Hilden, Germany) and further purified using an RNeasy Mini Kit (Qiagen) following the manufacturer's instructions. The concentration and purity of the extracted RNA were determined using a NanoDrop 2000 (Thermo Fisher Scientific, Wilmington, DE). At least two independent RNA extractions were performed for each sample. Total RNA (500 ng) was then reverse-transcribed into cDNA using a ReverTra Ace qPCR RT Kit (Toyobo, Osaka, Japan). Q-PCR analysis was performed using an MX3005P system (Agilent Technologies, Santa Clara, CA, USA) with THUNDERBIRD SYBR qPCR Mix (Toyobo) following the manufacturer's instructions. All Q-PCR reactions were performed in duplicate and the results were analyzed using the comparative Ct method. The Ct values of samples and controls were normalized to those of *Gapdh*. All primer sets used in this study are shown in Supplementary Table S1.

### Immunohistochemistry

Skin or tumor tissues were fixed for 24 h in 10% neutral-buffered formalin (Nacalai Tesque Inc., Kyoto, Japan), dehydrated, and embedded in paraffin. Sections (4-μm thick) were prepared for hematoxylin and eosin staining and immunohistochemistry. Sections were deparaffinized in xylene and rehydrated in a graded series of ethanol. For antigen retrieval, sections were immersed in Target Retrieval Solution pH 9 or pH 6 (Dako, Hamburg, Germany), and incubated for 40 min at 100°C in a water bath or heated for 15 min at 121°C by autoclaving. Non-specific binding was blocked with Protein Block Serum-Free (Dako) for 30 min at room temperature (RT). Sections were incubated with primary antibodies diluted in 1% BSA/PBS overnight at 4°C. The primary antibodies used in this study were: rabbit polyclonal anti-TGF-β1 (V) antibody (Santa Cruz Biotechnology, Santa Cruz, CA), rat monoclonal anti-F4/80 (BM8) antibody (eBioscience, San Diego, CA), rabbit polyclonal anti-Nrf2 (H-300) antibody (Santa Cruz Biotechnology), rabbit polyclonal anti-keratin 14 (Poly19053) antibody (BioLegend, San Diego, CA), rat monoclonal anti-mouse Ki-67 (TEC-3) antibody (Dako), rabbit monoclonal anti-cleaved caspase-3 (5A1E) antibody (Cell Signaling Technology, Danvers, MA), rabbit monoclonal anti-phospho-p53 (Ser15) (D4S1H) antibody (Cell Signaling Technology), and mouse monoclonal anti-phospho-histone H2AX (Ser139) (JBW301) antibody (EMD Millipore, Billerica, MA). Endogenous peroxidase was inhibited with 0.3% hydrogen peroxide in methanol for 15 min. The sections were incubated with the secondary antibodies EnVision+ System-HRP Labeled Polymer Anti-Rabbit or Anti-Mouse (Dako), or N-Histofine Simple Stain Mouse MAX PO (Rat) (Nichirei Bioscience, Tokyo, Japan) for 30 min at RT except for the sections incubated with anti-Ki-67 antibody, which were incubated with biotinylated secondary antibody followed by incubation with peroxidase-conjugated streptavidin (Nichirei Bioscience). Signals were visualized by the Liquid DAB+ Substrate-Chromogen System (Dako) with nuclear counterstaining using hematoxylin. To quantify the immunohistochemical staining in mouse epidermis, cells stained positive for each protein of interest were counted per field (dermis) or per length of basement membrane (BM).

### Western blot analysis

Skin or tumor samples were snap-frozen in liquid nitrogen, ground to a fine powder by Multi-Beads Shocker (Yasui Kikai, Inc.), lysed in SDS sample buffer and prepared as previously described [27]. Protein samples (30 μg) were subjected to SDS-polyacrylamide gel electrophoresis, and immunoblot analysis was performed as previously described [27]. The antibodies used in this study were: rabbit polyclonal anti-TGF-β1 (V) antibody (Santa Cruz Biotechnology), rabbit monoclonal anti-phospho-Smad2 (Ser465/467) (138D4) antibody (Cell Signaling Technology), rabbit monoclonal anti-Smad2 (D43B4) antibody (Cell Signaling Technology) and mouse monoclonal anti-β-actin (AC-74) antibody (Sigma-Aldrich, Saint Louis, MO). To confirm equal loading, membranes were reprobed with anti-β-actin antibody.

### Isolation and short-term culture of primary mouse keratinocytes

Dorsal skin was obtained from newborn mice and immersed in Dispase II solution, 2.5 U/ml Dispase II (Roche, Indianapolis, IN) with antibiotic-antimycotic solution CnT-ABM10 (CELLnTEC, Bern, Switzerland) in CnT-PR medium (CELLnTEC) for 16 h at 4°C. After being washed with PBS, the sheet of epidermis, separated from the dermis, was floated on a 500 μl drop of TrypLE select (Thermo Fisher Scientific) for 25 min at RT. After addition of 2 ml of medium to the drop, the sheet of epidermis was shaken gently on the drop to disperse the keratinocytes. The cell suspension was collected in a 15-ml tube containing fetal bovine serum (FBS). The cells were filtered through a 70-μm cell strainer to remove clumps and debris, centrifuged for 7 min at 200 × *g*, suspended in medium with 10% FBS and centrifuged again for 7 min at 1300 rpm. The cells were plated in CnT-PR medium with 10% FBS at 1.5 × 10^5^ cells/cm^2^ and incubated at 37°C in a humidified 5% CO_2_ incubator. The medium was removed 16 h after plating and replaced with CnT-PR medium without FBS. The medium was changed every other day.

### Cell proliferation and cytotoxic assay

Cell proliferation was measured by water-soluble tetrazolium (WST)-1 assay (Roche). Mouse keratinocytes were cultured at a density of 5.0 × 10^4^ cells/well in a 96-well microplate. At 0, 1, 2, and 4 days after seeding, the WST-1 reagent was added to the cultures and cells were incubated for 4 h. For cytotoxic assay, the cells were seeded at a density of 3.0 × 10^5^ cells/well in a 24-well microplate and incubated for 24 h. The cells were exposed for 24 h to DMBA (Sigma-Aldrich) or dimethyl sulfoxide (DMSO, Wako, Osaka, Japan) as a control, or exposed for 48 h to cisplatin (CDDP, Bristol-Myers Squibb, New York, NY) or PBS as a control. WST-1 reagents were added to the cultures and cells were incubated for 4 h before absorbance measurement. Absorbance at 450 nm and 650 nm was measured using a microplate reader, Powerscan 4 (DS Pharma Biomedical, Osaka, Japan). The measured absorbance (A450–A650) is directly proportional to the number of living cells. Note that the cells were pretreated with 10 ng/ml TGF-β1 (R&D Systems, Minneapolis, MN), 10 ng/ml SB431542 (TGFBRI inhibitor, Selleck Chemicals, Houston, TX) or PBS as a control for 24 h before incubation with DMBA.

### Immunofluorescence analysis

Keratinocytes isolated from newborn mice were grown in 3.5-cm glass-based dishes, washed with PBS and fixed with 4% paraformaldehyde for 15 min at RT. After washing with PBS, 0.3% Triton X-100 (Wako) in PBS was applied for 3 min at RT. The cells were blocked using 3% skim milk in PBS for 30 min and incubated for 1 h at RT with appropriate primary antibodies. The primary antibodies used in this study were: rabbit monoclonal anti-phospho-Smad2 (Ser465/467) (138D4) (Cell Signaling Technology), rat monoclonal anti-p21 [HUGO291] (Abcam, Cambridge, UK) and rabbit polyclonal anti-Nrf2 (H-300) (Santa Cruz Biotechnology). The cells were washed with PBS and incubated for 30 min at RT in PBS containing Alexa Fluor 488 and/or 555-conjugated secondary antibodies (Life Technologies, Carlsbad, CA) with nuclear counterstaining using DAPI. Immunofluorescence was detected with a confocal laser-scanning microscope (LSM 700, Carl Zeiss, Oberkochen, Germany). To analyze fluorescence intensity, *CD109^+/+^* and *CD109^−/−^* keratinocytes were immunostained at the same time and under the same conditions. The “Histogram” function of ZEN software (Carl Zeiss) was used to detect the fluorescence intensity per cell as follows: the fluorescence intensity for individual cells in the green or red channels was measured for the area inside the nuclei only, by masking the area outside the nuclei using the blue channel (DAPI). The average measurements, normalized to DAPI fluorescence intensity, were used to compare fluorescence intensity between *CD109^+/+^*and *CD109^−/−^* cells.

### Skin tumorigenesis assay

Murine skin tumors were induced by a standard two-stage chemical carcinogenesis protocol as previously described [49, 50]. In brief, 6–8-week-old female mice were shaved on the dorsal skin with electric clippers and received a single topical application of 100 nmol DMBA (Sigma-Aldrich) in 200 μl acetone 24 h after shaving. One week after DMBA treatment, the mice received topical applications of 5 nmol TPA (Sigma-Aldrich) in 200 μl acetone twice weekly for 15 weeks. Mice were assessed on weekly basis for a period of up to 25 or 35 weeks after DMBA treatment. The appearance of skin tumors was recorded once a week. All animal protocols were approved by the Animal Care and Use Committee of Nagoya University Graduate School of Medicine (Approval ID number: 27338).

### Analysis of H-*ras* mutations in codon 61

Genomic DNA was isolated from snap-frozen DMBA/TPA-treated skin using a NucleoSpin Tissue kit (Macherey-Nagel, Düren, Germany) following the manufacturer's instruction. The concentration and purity of the extracted genomic DNA were determined using a NanoDrop 2000 (Thermo Fisher Scientific). Mutation of H-*ras* in codon 61 was detected by mutation-specific PCR assay [36]. The primers for mutant H-*ras^(Q61L)^* were as follows: forward primer, 5′-CTA AGC CTG TTG TTT TGC AGG AC-3′ and reverse primer, 5′-CAT GGC ACT ATA CTC TTC TA-3′. Before amplification, genomic DNA samples were denatured for 5 min at 95°C. Each amplification cycle consisted of denaturation for 30 sec at 95°C, hybridization for 20 sec at 58°C, and elongation for 20 sec at 78°C. After 40 cycles of amplification, 5 μl of PCR products were analyzed by 1.5% agarose gel electrophoresis and ethidium bromide staining.

### Statistical analysis

Statistical analyses were performed using IBM SPSS software 22.0 (IBM, Armonk, NY). Numerical results are presented as mean value ± standard deviation (S.D.) or standard error of the mean (S.E.M.), or in box and whisker plots. For comparison of the differences between two groups, a two-tailed Student's t test, Mann-Whitney U test or one-way analysis of variance (ANOVA) was performed. Two-way ANOVA was used to evaluate differences to compare four groups. For tumor-free analysis, Kaplan-Meier log-rank analysis was performed. Clinicopathological features were evaluated with χ^2^ tests. A P value of < 0.05 was considered statistically significant.

## SUPPLEMENTARY DATA




