# Supplementary material for: Suppression of skin tumorigenesis in CD109-deficient mice
Source: Oncotarget. 2016 Oct 14;7(50):82836–50. doi: 10.18632/oncotarget.12653 (PMC5347736; doi:10.18632/oncotarget.12653)
Supplement: Supplementary file 1 [file oncotarget-07-82836-s001.pdf]

# Suppression of skin tumorigenesis in CD109-deficient mice

## SUPPLEMENTARY DATA

## SUPPLEMENTARY METHODS

### Skin inflammation assay

The dorsal skin of 6–8-week-old female mice was shaved with electric clippers, and received a first topical application of 8.1 nmol TPA (Sigma-Aldrich) in 200 μl acetone 48 h after being shaved. The mice were treated with TPA a further five times at 48 h intervals. Acetone-treated mice were used as controls. Mice were sacrificed and assessed 48 h after the final TPA treatment.

### Immunohistochemistry

The primary antibodies used in the supplementary immunohistochemistry experiments were: rabbit polyclonal anti-TGFBRI (V-22) and anti-TGFBRII (C-16) antibodies (Santa Cruz Biotechnology); mouse monoclonal anti-CD109 (C-9) and anti-p21 (F-5) antibodies (Santa Cruz Biotechnology); rabbit monoclonal anti-TNF-α (D2D4) and anti-pSMAD2 (138D4) antibodies (Cell Signaling Technology); and rabbit polyclonal anti-GM-CSF (ag12142) antibody (Proteintech Group Inc., Rosemont, IL).

### Clodronate treatment

Clophosome-A clodronate liposomes (clodronate liposomes) and placebo control liposomes for Clophosome-A (control liposomes) were purchased from FormuMax Scientific Inc. (Sunnyvale, CA). Liposomes (200 μL) were subcutaneously injected into 6–8-week-old female mice on days 0, 2 and 4 of the experiment. Mice were sacrificed 14 days after the first application.

### Western blot analysis

The primary antibodies used in the supplementary western blotting experiments were: rabbit monoclonal anti-Gsta3 antibody (Abcam), rabbit polyclonal anti-Slc7a11 antibody (Abcam), mouse monoclonal anti-Nqo1(A180) antibody (Santa Cruz Biotechnology) and rabbit polyclonal anti-Cbr1 antibody (Abcam).

### RNA interference

For transient silencing of p21, keratinocytes isolated from newborn *CD109*$^{+/+}$ and *CD109*$^{-/-}$ mice were transfected using Lipofectamine RNAiMAX transfection reagent (Thermo Fisher Scientific) according to the manufacturer's protocols with ON-TARGETplus Non-targeting Control Pool or ON-TARGETplus Mouse Cdkn1a (12575) siRNA-SMARTpool (GE Healthcare, Lafayette, CO) at a final concentration of 10 nM.

### DNA microarray analysis

*CD109*$^{+/+}$ and *CD109*$^{-/-}$ mice were treated with DMBA, and sacrificed 24 h after the treatment. Total RNA was extracted from the skins of the untreated and DMBA-treated *CD109*$^{+/+}$ and *CD109*$^{-/-}$ mice using TRIzol (Thermo Fisher Scientific) and an RNeasy Mini Kit (Qiagen) (n = 2 per group; 8 mice in total). Cyanine-3 (Cy3)-labeled cRNA was prepared from 100 ng total RNA using a Low Input Quick Amp Labeling Kit, One-Color (Agilent Technologies). Fragmentation of Cy3-labeled cRNA and hybridization to SurePrint G3 Mouse GE 8×60K Microarray were performed by CDM Center (Takara Bio Inc., Kusatsu, Japan). The data have been deposited in the Gene Expression Omnibus (GEO; accession number GSE87390).

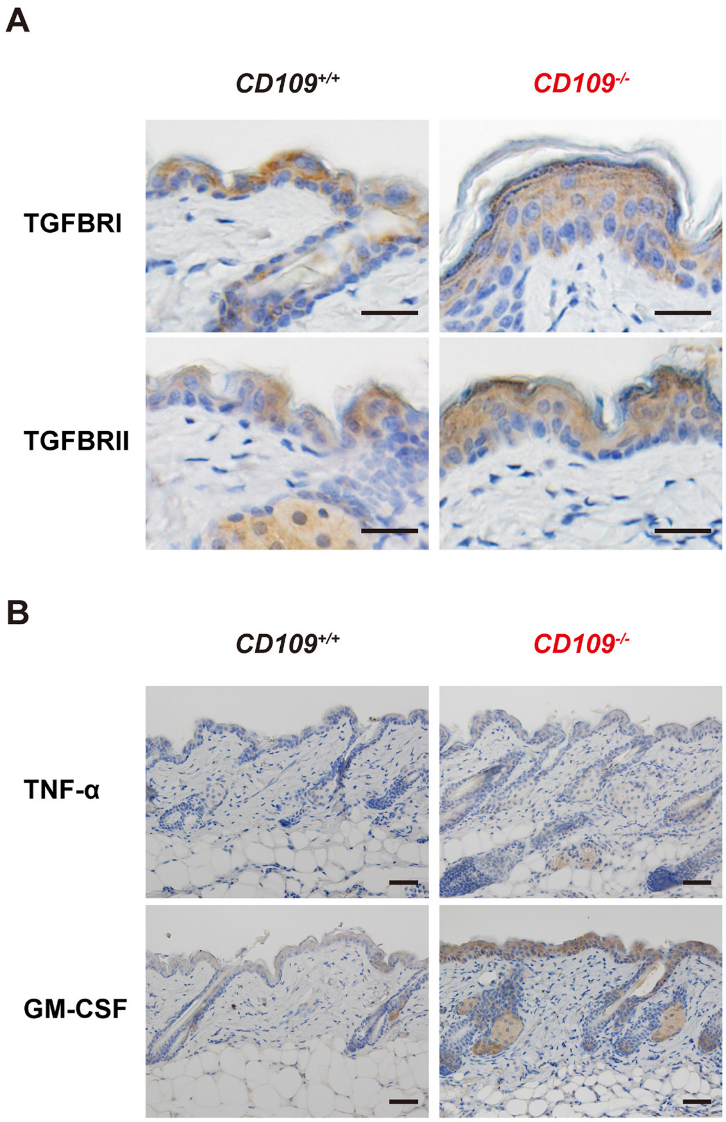


**Supplementary Figure S1: Immunohistochemical analyses of *CD109*$^{+/+}$ and *CD109*$^{-/-}$ skin. A.** Skin of 6-week-old *CD109*$^{+/+}$ and *CD109*$^{-/-}$ mice immunostained using anti-TGFBRI or TGFBRII antibodies. Scale bars, 25 µm. **B.** *CD109*$^{+/+}$ and *CD109*$^{-/-}$ skins immunostained using anti-TNF-α or GM-CSF antibodies. Scale bars, 50 µm.

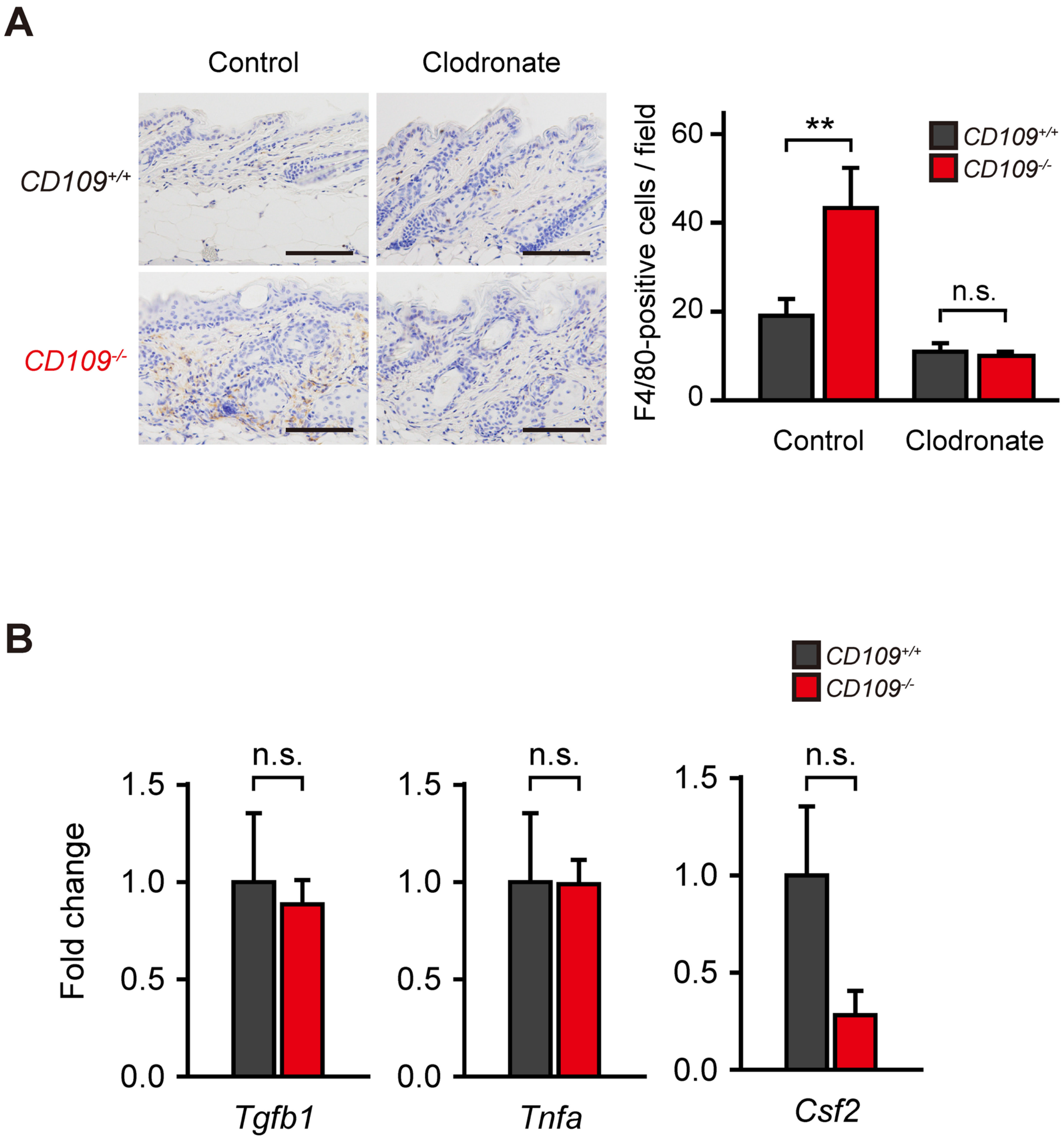


**Supplementary Figure S2: Macrophage depletion decreased the difference in *Tgfb1* expression levels between *CD109*$^{+/+}$ and *CD109*$^{-/-}$ skin. A.** Left, representative microscopic images of *CD109*$^{+/+}$ and *CD109*$^{-/-}$ skins treated with control or clodronate liposomes. Scale bars, 100 µm. Right, quantification of F4/80-positive cells in *CD109*$^{+/+}$ and *CD109*$^{-/-}$ skins (n = 4 per group). **B.** Expression levels of mRNAs encoding *Tgfb1*, *Tnfa* and *Csf2* determined by quantitative PCR in *CD109*$^{+/+}$ and *CD109*$^{-/-}$ skins treated with clodronate liposomes (n = 4 per group). n.s., not significant; $^{**}P < 0.01$.

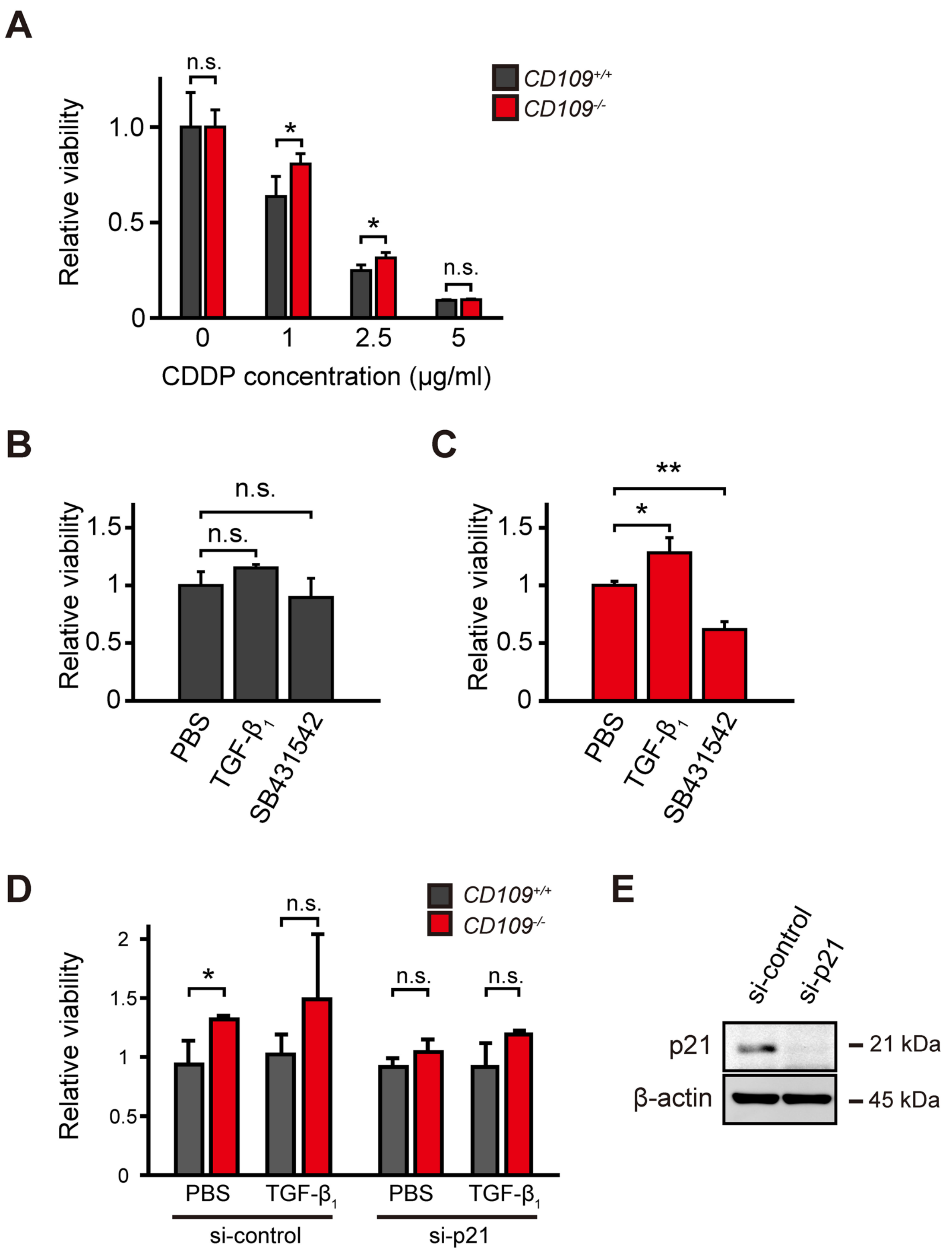


**Supplementary Figure S3: Susceptibility of primary mouse keratinocytes to CDDP-induced apoptosis. A.** Viability of *CD109*$^{+/+}$ and *CD109*$^{-/-}$ keratinocytes measured by WST-1 assay in the presence of CDDP (n = 3 per group). **B, C.** Viability of *CD109*$^{+/+}$ (B) and *CD109*$^{-/-}$ (C) keratinocytes measured by WST-1 assay in the presence of DMBA (20 μg/ml, n = 3 per group). Cells were pretreated with PBS only (control), TGF-$\beta_1$ (10 ng/ml) or SB431542 (TGFBRI inhibitor, 10 ng/ml) for 24 h before incubation with DMBA. **D.** Viability of *CD109*$^{+/+}$ and *CD109*$^{-/-}$ keratinocytes transfected with siRNA targeting *p21*, measured by WST-1 assay in the presence of DMBA (10 μg/ml, n = 3 per group). Cells were pretreated with PBS only (control) or TGF-$\beta_1$ (10 ng/ml) for 24 h before incubation with DMBA. **E.** Effects of siRNA on p21 expression in keratinocytes. CDDP, cisplatin; n.s., not significant; $*P < 0.05$; $**P < 0.01$.

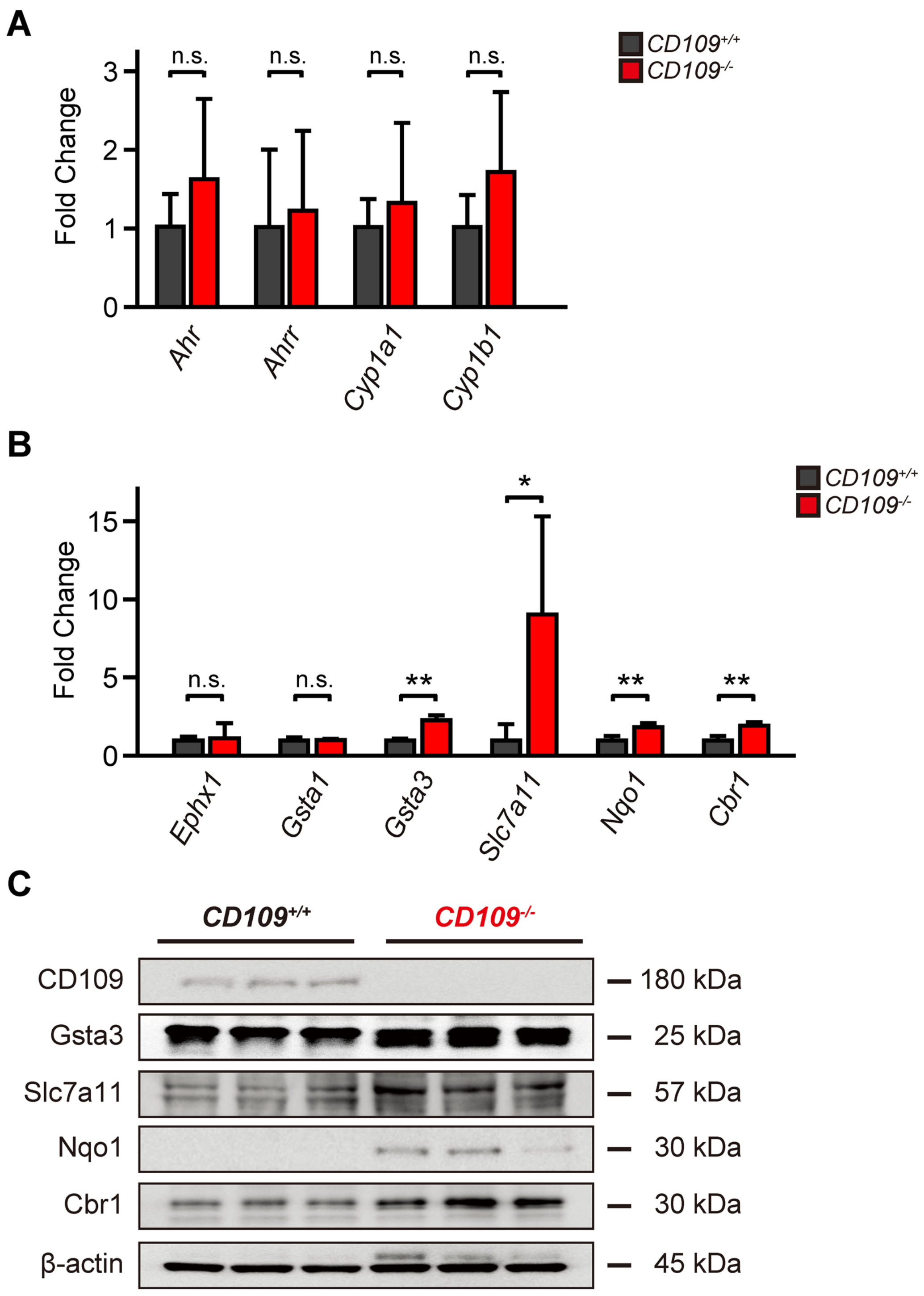


**Supplementary Figure S4: CD109 deficiency did not affect the expression of detoxification enzymes that are not regulated by Nrf2, but increased the expression of several detoxification enzymes that are regulated by Nrf2 in mouse skin. A, B.** Expression levels of mRNAs encoding the indicated detoxification enzymes, which are not regulated by Nrf2 (A) and regulated by Nrf2 (B), in the *CD109*$^{+/+}$ and *CD109*$^{-/-}$ skins (n = 4 per group), determined by quantitative PCR. Values are normalized to *CD109*$^{+/+}$ levels. **C.** Immunoblot analysis of whole-skin lysates from *CD109*$^{+/+}$ and *CD109*$^{-/-}$ mice using antibodies against CD109, Gsta3, Slc7a11, Nqo1 and Cbr1 (n = 3 per group). Expression of β-actin is shown as a loading control. n.s., not significant; **P* < 0.05; ***P* < 0.01.

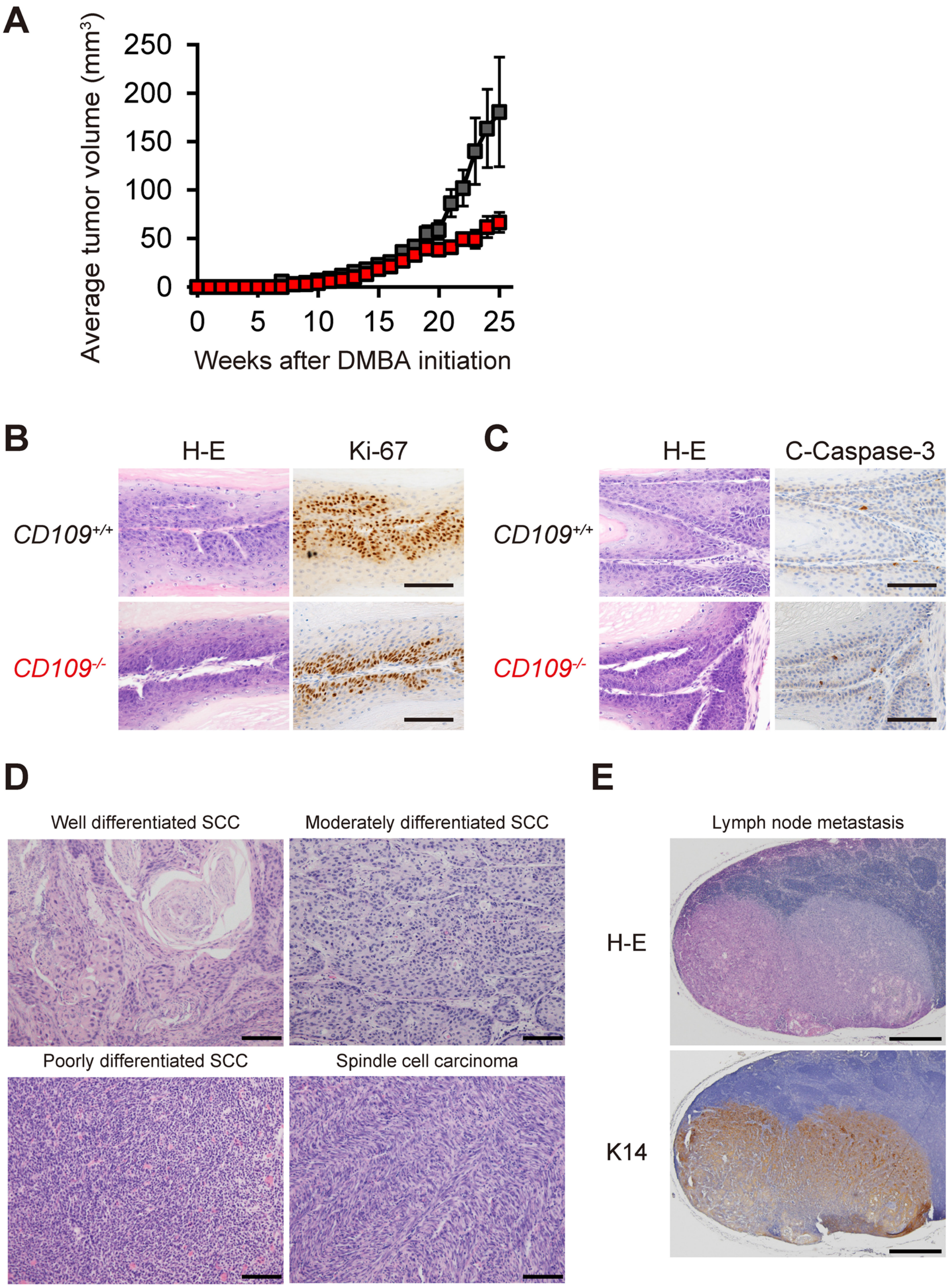


**Supplementary Figure S5: DMBA/TPA-induced skin tumors in *CD109*$^{-/-}$ mice were smaller than in *CD109*$^{+/+}$ mice, and similar histologically to those in *CD109*$^{+/+}$ mice. A.** Average tumor volume was decreased in *CD109*$^{-/-}$ mice compared with *CD109*$^{+/+}$ mice (n = 9 per group). **B, C.** Immunohistochemical staining for Ki-67 (B) and cleaved caspase-3 (C) and corresponding hematoxylin and eosin staining in *CD109*$^{+/+}$ and *CD109*$^{-/-}$ papillomas at 17 weeks after DMBA initiation. Scale bars, 100 µm. **D.** Representative images of well, moderately, and poorly differentiated SCCs and spindle cell carcinoma in our carcinogenesis model at 25 weeks after DMBA initiation. Scale bars, 100 µm. **E.** Representative image of lymph node metastatic lesions. Hematoxylin and eosin staining (top) and keratin 14 immunostaining (bottom). SCC was positive for keratin 14. Scale bars, 500 µm. SCC, squamous cell carcinoma; K14, keratin 14.

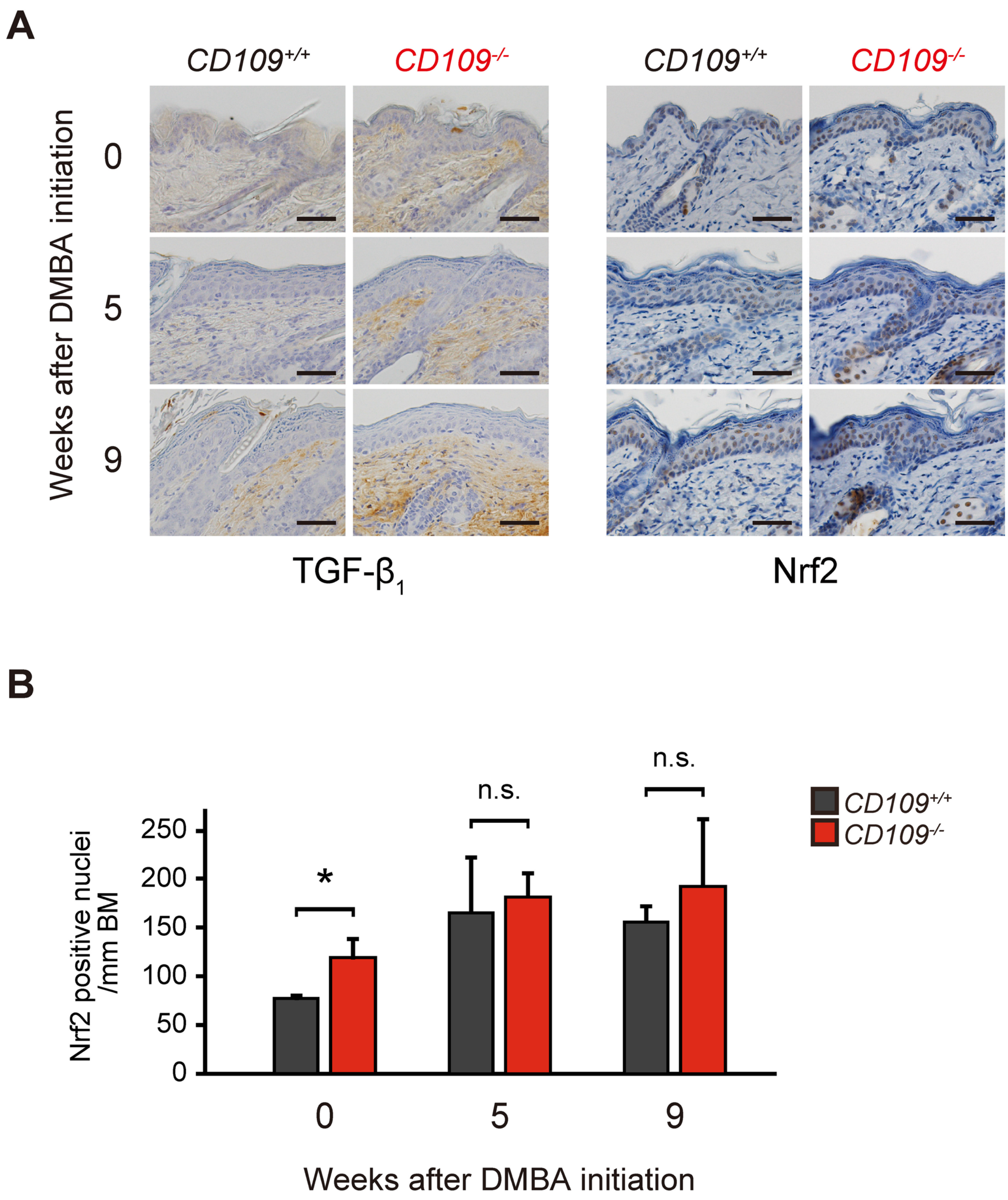


**Supplementary Figure S6: TGF-β1 and Nrf2 expression in *CD109*$^{+/+}$ and *CD109*$^{-/-}$ skin before papilloma formation. A.** Immunohistochemical staining using antibodies against TGF-β1 (left) and Nrf2 (right) in *CD109*$^{+/+}$ and *CD109*$^{-/-}$ skins at 0 (no treatment), 5, and 9 weeks after DMBA initiation. Scale bars, 50 μm. **B.** Number of Nrf2-positive cells in epidermis per mm of basement membrane (BM) at 0, 5 or 9 weeks after DMBA initiation (n = 3 per group). *$P < 0.05$; n.s., not significant.

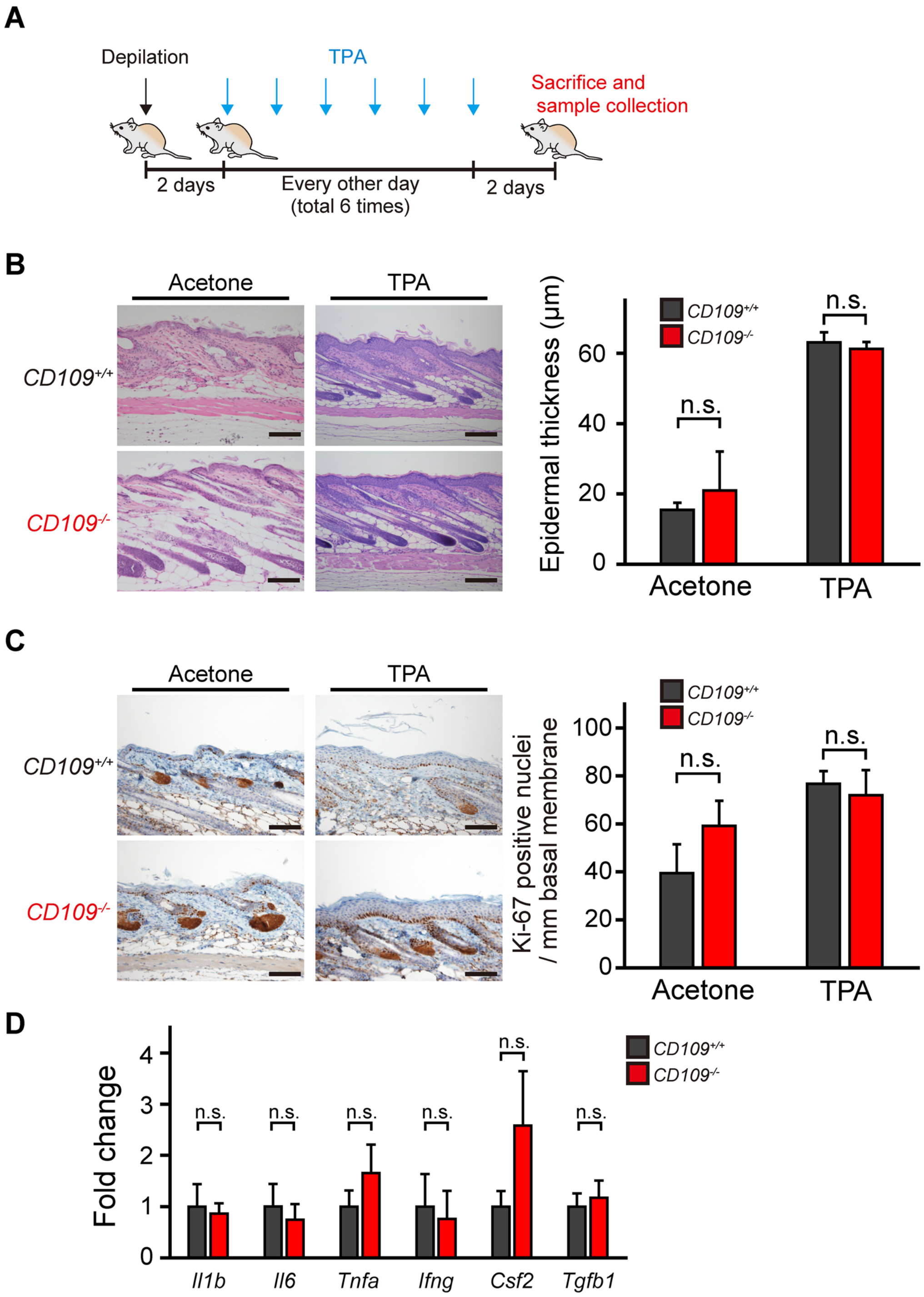


**Supplementary Figure S7: CD109 deficiency did not promote TPA-induced epidermal hyperplasia. A.** Schematic of protocol for induction of cutaneous inflammation with TPA. **B.** Left, representative microscopic images of *CD109*$^{+/+}$ and *CD109*$^{-/-}$ skins treated with acetone only (control) or TPA. Right, quantification of epidermal thickness in *CD109*$^{+/+}$ and *CD109*$^{-/-}$ skins (n = 3 per group). Scale bars, 200 µm. **C.** Left, immunohistochemical staining for Ki-67 in *CD109*$^{+/+}$ and *CD109*$^{-/-}$ skins treated with acetone only (control) or TPA. Right, number of Ki-67-positive cells in epidermis per mm of basement membrane (right graph; n = 3 per group). Scale bars, 200 µm. **D.** Expression levels of mRNAs encoding the indicated cytokines in *CD109*$^{+/+}$ and *CD109*$^{-/-}$ skins treated with TPA (n = 3 per group), determined by quantitative PCR. Values are normalized to *CD109*$^{+/+}$ levels. n.s., not significant.

**Supplementary Table S1: List of primers used for quantitative PCR in this study**

| | Primer Sequences | |
|---|---|---|
| **Gene** | **Forward (5' →3')** | **Reverse (3' →5')** |
| *Il1b* | AAGCCTCGTGCTGTCGGACC | CAGGGTGGGTGTGCCGTCTT |
| *Il2* | TGCCCAAGCAGGCCACAGAA | AGTCCACCACAGTTGCTGACTCAT |
| *Il4* | GGGCTTCCAAGGTGCTTCGC | GCATGATGCTCTTTAGGCTTTCCAGG |
| *Il6* | GACAAAGCCAGAGTCCTTCAGAG | CTAGGTTTGCCGAGTAGATCTC |
| *Il10* | TGGTAGAAGTGATGCCCCAGGC | CACCTGCTCCACTGCCTTGC |
| *Tnfa* | CATCTTCTCAAAATTCGAGTGACAA | TGGGAGTAGACAAGGTACAACCC |
| *Ifng* | GCTGTTACTGCCACGGCACA | GGCTCTGCAGGATTTTCATGTCACC |
| *Csf2* | GATATTCGAGCAGGGTCTAC | GGGATATCAGTCAGAAAGGTT |
| *Tgfb1* | GCAACAATTCCTGGCGTTACC | CCCTGTATTCCGTCTCCTTGGT |
| *Tgfb2* | TCCCGAATAAAAGCGAAGAGC | GGTGCCATCAATACCTGCAAA |
| *Tgfb3* | CCAGATACTTCGACCGGATGA | TGACATCGAAAGACAGCCATTC |
| *Ahr* | AGGACCAAACACAAGCTAGA | TGGAGATCTCGTACAACACA |
| *Ahrr* | GCCAATGCTGTCTAATGAAG | AACAGAGCACCAAGAAAACA |
| *Cyp1a1* | CAGACCTCAGCTGCCCTATC | CTGCCACTGGTTCACAAAGA |
| *Cyp1b1* | CTTCGGAGCCAGCCAGGACA | ATGGCAGGTTGGGCTGGTCA |
| *Ephx1* | GACCCCTCTCCTGGGCCAAC | AGATGTAGGCAGCCAGGCCC |
| *Gsta1* | ACAGGCTGACCAGGGTGGAC | TGGCTGCCAGGCTGTAGGAAC |
| *Gsta3* | CCAGGAACCGTTACTTCCCTGCC | GGGGAGGTTGCTGACTCTGCT |
| *Slc7a11* | GCTGGCAGTCGCAGGACTGAT | GCAGGGACCCCAGTCAAGGT |
| *Nqo1* | ATCACCACTGGGGGTAGCGG | CTGCATGCGGGCATCTGGTG |
| *Cbr1* | TGACGACACCCCCTTCCACA | CCCCACCAGCTCCTCCTCTG |

**Supplementary Table S2: List of genes downregulated (each log2 ratio < −1) in *CD109*$^{+/+}$ skins and upregulated (each log2 ratio > 1) in *CD109*$^{-/-}$ skins with DMBA treatment compared with untreated skins**

| | | Mean log2 ratio | |
|---|---|---|---|
| **Probe ID** | **Gene Symbol** | ***CD109*$^{+/+}$** | ***CD109*$^{-/-}$** |
| A_55_P2007919 | ***Akr1c19*** | -4.26 | 3.41 |
| A_55_P2146837 | *Olfr481* | -1.61 | 2.27 |
| A_52_P469789 | *Calcr* | -1.92 | 2.15 |
| A_55_P2028004 | *Tas2r124* | -1.73 | 2.09 |
| A_51_P402443 | *Olfr836* | -1.76 | 2.01 |
| A_55_P2036620 | *Lrif1* | -1.77 | 1.87 |
| A_55_P1969960 | *Defa25* | -1.90 | 1.83 |
| A_51_P293015 | *Il25* | -1.89 | 1.70 |
| A_55_P2178818 | *Itk* | -1.91 | 1.60 |
| A_51_P220837 | *Olfr786* | -2.12 | 1.42 |
| A_51_P127738 | *Scn2a1* | -1.55 | 1.41 |
| A_55_P2009752 | *Hlf* | -1.59 | 1.20 |

The gene in bold is discussed in the text

**Supplementary Table S3: List of genes upregulated (each log2 ratio > 1) in *CD109*$^{+/+}$ skins and downregulated (each log2 ratio < −1) in *CD109*$^{-/-}$ skins with DMBA treatment compared with untreated skins**

| Probe ID | Gene Symbol | Mean log2 ratio | |
|---|---|---|---|
| | | ***CD109***$^{+/+}$ | ***CD109***$^{-/-}$ |
| A_51_P339793 | *Il1rl1* | 4.67 | -1.66 |
| A_52_P8459 | ***Tpsab1*** | 2.66 | -1.60 |
| A_55_P2019557 | *Mrgpra2b* | 5.12 | -1.39 |

The gene in bold is discussed in the text

**Supplementary Table S4: Pathological analysis of skin tumors from *CD109*$^{+/+}$ and *CD109*$^{-/-}$ mice at 40 weeks after DMBA initiation**

| ***CD109* genotypes** | +/+ | −/− | ***P* value** |
|---|---|---|---|
| Number of mice | 17 | 12 | |
| Total number of tumors | 590 | 396 | |
| Total number of invasive tumors | 52 | 36 | |
| Malignant conversion rate[a)] | 8.9% | 9.3% | 0.11 |
| **Primary skin lesions** | | | |
| Invasive tumor size (μ;m) | 9.2 ± 0.9 | 7.3 ± 0.8 | 0.11 |
| Differentiation | | | 0.17 |
| Conventional SCCs[b)] | | | |
| Well differentiated | 5 (10%) | 7 (20%) | |
| Moderately differentiated | 25 (48%) | 14 (39%) | |
| Poorly differentiated | 7 (13%) | 3 (8%) | |
| Spindle cell carcinoma | 15 (29%) | 12 (33%) | |
| Invasion depth | | | 0.42 |
| Dermis | 8 (15%) | 9 (25%) | |
| Hypodermis | 25 (48%) | 8 (22%) | |
| Muscle | 13 (25%) | 17 (47%) | |
| Striated muscle | 6 (12%) | 2 (6%) | |
| **Metastases** | | | 0.26 |
| Metastatic lesions | 11/17 (64%) | 10/12 (83%) | |
| Lymph node | 11 (64%) | 10 (83%) | |
| Lung | 8 (47%) | 5 (42%) | |
| Spleen | 4 (24%) | 4 (33%) | |
| Pleural metastasis | 1 (6%) | 1 (8%) | |
| Liver | 1 (6%) | 0 (0%) | |
| Heart (Tumor embolism) | 1 (6%) | 0 (0%) | |

a) Malignant conversion rate = total number of invasive tumors/total number of tumors
b) SCCs, squamous cell carcinomas

**Supplementary Table S5: List of genes downregulated (each log2 ratio < −4) or upregulated (each log2 ratio > 4) in *CD109*$^{-/-}$ skins compared with in *CD109*$^{+/+}$ skins.** Genes in bold are discussed in the text

See Supplementary File 1
