# Supplementary material for: Suppression of skin tumorigenesis in CD109-deficient mice
Source: Oncotarget. 2016 Oct 14;7(50):82836–50. doi: 10.18632/oncotarget.12653 (PMC5347736; doi:10.18632/oncotarget.12653)
Supplement: Supplementary file 2 [file oncotarget-07-82836-s002.docx]

**Supplementary Table S5**

List of genes downregulated (each log2 ratio < −4) or upregulated (each log2 ratio > 4) in *CD109^-/-^* skins compared with in *CD109^+/+^* skins. Genes in bold are discussed in the text.

| Probe ID | Gene Symbol | Mean log2 ratio |
| --- | --- | --- |
| A_51_P349546 | *Cd109* | -9.10 |
| A_52_P21486 | *Hamp2* | -7.73 |
| A_51_P369762 | *Nmrk2* | -5.25 |
| A_55_P2088550 | *Rpl10l* | -4.60 |
| A_51_P441898 | *Krt25* | 15.22 |
| A_51_P284503 | *Krtap22-2* | 13.19 |
| A_51_P336049 | *Krt33b* | 12.93 |
| A_51_P364639 | *Krt71* | 12.84 |
| A_52_P321831 | *Krtap3-1* | 12.26 |
| A_51_P380991 | *Krt34* | 12.09 |
| A_55_P2095054 | *Krtap6-1* | 12.08 |
| A_55_P2086334 | *Krt85* | 12.03 |
| A_51_P386688 | *Krtap19-9b* | 11.80 |
| A_55_P2151225 | *Krtap16-1* | 11.80 |
| A_51_P412926 | *Krt27* | 11.79 |
| A_51_P221823 | *Krtap21-1* | 11.75 |
| A_55_P1968799 | *Krtap1-5* | 11.71 |
| A_51_P135118 | *Krtap13-1* | 11.50 |
| A_51_P104681 | *Krtap6-5* | 11.47 |
| A_51_P100624 | *Krtap9-3* | 11.46 |
| A_55_P1985294 | *Krtap5-5* | 11.20 |
| A_51_P345995 | *Krtap19-3* | 11.04 |
| A_51_P335694 | *Krtap4-7* | 11.02 |
| A_51_P172424 | *Krtap4-16* | 11.02 |
| A_55_P2092475 | *Krtap20-2* | 10.91 |
| A_55_P2160029 | *Krtap7-1* | 10.62 |
| A_51_P204350 | *Krt33a* | 10.32 |
| A_55_P2007085 | *Krtap19-9b* | 10.24 |
| A_55_P2140022 | *Krt35* | 10.15 |
| A_55_P1985648 | *Krtap6-2* | 10.04 |
| A_55_P1966450 | *Krt83* | 9.99 |
| A_51_P132400 | *Krtap14* | 9.97 |
| A_52_P131062 | *Krtap8-1* | 9.73 |
| A_52_P463962 | *Krtap16-3* | 9.66 |
| A_55_P2006812 | *Krtap11-1* | 9.63 |
| A_51_P408199 | *Krtap4-2* | 9.49 |
| A_55_P2147836 | *Lyg2* | 9.47 |
| A_51_P450549 | *Padi3* | 9.39 |
| A_66_P114558 | *Krtap1-3* | 9.35 |
| A_55_P2098150 | *Krtap5-5* | 9.29 |
| A_55_P2145611 | *Krt86* | 9.15 |
| A_55_P2154987 | *Krtap5-2* | 9.12 |
| A_55_P2403159 | *Fam26d* | 8.93 |
| A_51_P468456 | *S100a3* | 8.91 |
| A_52_P468068 | *Tchh* | 8.77 |
| A_55_P2038242 | *Tchhl1* | 8.71 |
| A_55_P2151178 | *Krtap19-1* | 8.50 |
| A_51_P498429 | *Krtap15* | 8.45 |
| A_51_P475816 | *Krtap2-4* | 8.44 |
| A_55_P2009001 | *Gprc5d* | 8.35 |
| A_55_P2005525 | *Krtap12-1* | 8.33 |
| A_55_P2158067 | *Krtap4-16* | 8.28 |
| A_55_P1976972 | *Krt28* | 8.24 |
| A_55_P2027213 | *Krtap20-2* | 8.19 |
| A_51_P501873 | *Krt26* | 8.08 |
| A_55_P2172096 | *Mc1r* | 7.86 |
| A_55_P2136606 | *Crnn* | 7.69 |
| A_55_P2140057 | *Krt32* | 7.65 |
| A_55_P2032930 | *Krtap3-3* | 7.41 |
| A_55_P2110698 | *Krtap4-9* | 7.40 |
| A_55_P2060011 | *Krtap5-5* | 7.39 |
| A_55_P2162379 | *Actbl2* | 7.35 |
| A_51_P234359 | *Sct* | 7.32 |
| A_55_P2114308 | *Krt40* | 7.23 |
| A_55_P1973970 | *Krt73* | 7.23 |
| A_55_P2009732 | *Prr9* | 7.19 |
| A_51_P338031 | *Trpm1* | 7.12 |
| A_55_P2140042 | *Krt31* | 7.11 |
| A_55_P1964672 | *Krt28* | 7.08 |
| A_55_P2028873 | *Dsg4* | 7.05 |
| A_55_P1985298 | *Krtap5-2* | 6.98 |
| A_51_P189899 | *Olfr1134* | 6.97 |
| A_55_P2070940 | *Krtap20-2* | 6.96 |
| A_55_P2139703 | *Krtap5-4* | 6.94 |
| A_55_P2116435 | *Gpr143* | 6.86 |
| A_51_P474701 | *Fbp1* | 6.85 |
| A_52_P49014 | ***Shh*** | 6.79 |
| A_55_P2042161 | *Krtap26-1* | 6.77 |
| A_51_P370458 | *Krtap17-1* | 6.75 |
| A_55_P2140571 | *Krtap24-1* | 6.71 |
| A_51_P480202 | *Dlx2* | 6.70 |
| A_66_P135018 | *Krtap5-1* | 6.63 |
| A_55_P2043777 | *Trpm1* | 6.62 |
| A_55_P2088755 | *Krtap4-1* | 6.57 |
| A_55_P2124233 | *Vsig8* | 6.51 |
| A_51_P468073 | *Ggt1* | 6.50 |
| A_55_P2093569 | *Slc45a2* | 6.49 |
| A_55_P2081283 | *Dlx4* | 6.38 |
| A_55_P2145626 | *Krt82* | 6.33 |
| A_55_P2006311 | *Krtap9-1* | 6.27 |
| A_55_P2111172 | *Mecom* | 6.23 |
| A_55_P2065159 | *Krtap9-5* | 6.20 |
| A_51_P162144 | *Oca2* | 6.16 |
| A_52_P63855 | ***S100a7a*** | 6.09 |
| A_51_P205385 | *Uox* | 5.93 |
| A_55_P2017347 | *Krtap11-1* | 5.91 |
| A_52_P265937 | *Tagln3* | 5.89 |
| A_55_P2173768 | *Pkhd1* | 5.76 |
| A_55_P2052145 | *Ly6g6d* | 5.70 |
| A_52_P534583 | *Ahsp* | 5.47 |
| A_51_P389396 | *Gykl1* | 5.45 |
| A_55_P2041828 | *Tubb3* | 5.45 |
| A_51_P454873 | *Npy* | 5.40 |
| A_51_P135268 | *Corin* | 5.37 |
| A_55_P2043486 | *Msx2* | 5.35 |
| A_52_P90805 | *Lrrc15* | 5.34 |
| A_55_P2178698 | *Gprc5d* | 5.34 |
| A_52_P382886 | *Gjb2* | 5.21 |
| A_55_P1973945 | *Krt75* | 5.20 |
| A_51_P283968 | *Adamts18* | 5.12 |
| A_52_P69020 | *Slc24a5* | 5.04 |
| A_51_P222337 | *Rspo2* | 5.04 |
| A_52_P151393 | *Lrrc75b* | 5.01 |
| A_55_P2106335 | *Sstr3* | 4.97 |
| A_52_P464831 | *Dlx1* | 4.92 |
| A_51_P208145 | *Pmel* | 4.92 |
| A_55_P2083307 | *Capn8* | 4.92 |
| A_52_P536494 | *Mycn* | 4.84 |
| A_55_P2118996 | *Foxe1* | 4.83 |
| A_51_P158545 | *Ankk1* | 4.78 |
| A_55_P2054261 | *C2cd4b* | 4.74 |
| A_52_P31381 | *Slc26a3* | 4.70 |
| A_55_P2104219 | *Hoxc13* | 4.67 |
| A_51_P491378 | *Csn1s1* | 4.63 |
| A_55_P2054027 | *Chst5* | 4.61 |
| A_51_P176474 | *Kndc1* | 4.53 |
| A_51_P331044 | ***Il20*** | 4.46 |
| A_55_P1953402 | *Mlana* | 4.44 |
| A_55_P2100968 | *Dnah7b* | 4.40 |
| A_55_P2133007 | *Cntfr* | 4.38 |
| A_55_P2070045 | *Golga7b* | 4.38 |
| A_52_P263658 | *Hes2* | 4.34 |
| A_66_P119810 | *H2afb1* | 4.22 |
